# New Sights into Bioinformatics of Gene Regulations and Structure

**DOI:** 10.3390/ijms26136442

**Published:** 2025-07-04

**Authors:** Anastasia A. Anashkina, Nina G. Orlova, Nikolay A. Kolchanov, Yuriy L. Orlov

**Affiliations:** 1 Engelhardt Institute of Molecular Biology, Russian Academy of Sciences, 119991 Moscow, Russia; 2 The Digital Health Center, I.M. Sechenov First Moscow State Medical University of the Ministry of Health of the Russian Federation (Sechenov University), 119991 Moscow, Russia; 3 Department of Mathematics, Financial University Under the Government of the Russian Federation, 125167 Moscow, Russia; ngorlova@fa.ru; 4 Institute of Cytology and Genetics, Siberian Branch of the Russian Academy of Sciences, 630090 Novosibirsk, Russia; 5 Department of Natural Sciences, Novosibirsk State University, 630090 Novosibirsk, Russia; 6 Agrarian and Technological Institute, Peoples’ Friendship University of Russia, 117198 Moscow, Russia

This article overviews recent findings on molecular mechanisms of gene expression regulation published in the “New Sights into Bioinformatics of Gene Regulations and Structure” (https://www.mdpi.com/journal/ijms/special_issues/MVA479KFR7, accessed on 30 June 2025) Special Issue. Key approaches are based on bioinformatics methods, high-throughput sequencing, and post-genome technologies. We have initiated this Special Issue based on the discussions on bioinformatics of gene expression regulation at the transcription level presented at the “Bioinformatics of Genome Regulation and Structure/Systems Biology” (BGRS/SB) conference series in Novosibirsk, Russia. Begun in 1998, BGRS/SB is the longest-running biannual conference series gathering scientists, bioinformaticians, medical doctors, and geneticists in Novosibirsk, Russia (https://bgrssb.icgbio.ru/2024/, accessed on 30 June 2025). IJMS has already published several successful Special Issues on the bioinformatics of gene expression, including the following: “Molecular Mechanisms of Gene Expression: ‘Bioinformatics of Gene Regulations and Structure’” (2020), “Bioinformatics of Gene Regulations and Structure—2022” (https://www.mdpi.com/journal/ijms/special_issues/Bioinformatics_Gene, accessed on 30 June 2025), and “25 Anniversary of Bioinformatics of Genome Regulation and Structure Conference Series” (https://www.mdpi.com/journal/ijms/special_issues/0LGA6103S5, accessed on 30 June 2025), as well as earlier Special Issues [1,2]. The BGRS conference continues initiating series of science discussions on perspective research fields such as interdisciplinary studies in computational genomics of model organisms—plants, animals, bacteria, complex network interactions, and the systems biology approaches for gene transcription analysis, including machine learning and AI (artificial intelligence) tools [3].

We have previously organized similar Special Issues on the bioinformatics of gene expression regulation at Frontiers in Genetics (https://www.frontiersin.org/research-topics/40408/bioinformatics-of-genome-regulation-and-systems-biology-volume-iii, https://www.frontiersin.org/research-topics/21036/high-throughput-sequencing-based-investigation-of-chronic-disease-markers-and-mechanisms, accessed on 30 June 2025) and another series with BioMedCentral publisher (https://bmcgenomics.biomedcentral.com/articles/supplements/volume-20-supplement-3, accessed on 30 June 2025). This year, we continue the series of Special Issues in the Gene Expression journal (https://www.xiahepublishing.com/journal/ge/features/bioinformatics, accessed on 30 June 2025).

The key point of this Special Issue is the regulation of gene expression at the transcriptional level. Contemporary methods here include machine learning, data integration, and AI applications [4]. The articles in the current Special Issue could be classified by methods (neural networks, Alphafold, microRNA prediction) as well as by area of the study—cell lines, domestic animals, fungi, and plants.

We open this collection of papers with AI applications. Shoryu Teragawa and co-authors (Contribution 1) presented a deep learning model for DNA methylation prediction, called DeepPGD (https://doi.org/10.3390/ijms25158146). Prediction of epigenetic modification sites is important for gene expression analysis. The series of algorithms for the BiLSTM (bidirectional long short-term memory) model continues this perspective field [5,6]. BiLSTM helps to effectively extract intricate DNA structural and sequence features. Such AI models were recently applied to effectively capture DNA-sparse higher-order sequence features [6].

Kohei Uemura and Takashi Ohyama (https://doi.org/10.3390/ijms25031487) presented a fundamental study on physical peculiarities around transcription start sites and a site corresponding to the TATA box (Contribution 2). This topic has been highlighted in a series of works on TATA box-binding mechanisms [7,8]. The latest model uses SNPs (single nucleotide polymorphisms) in the TATA box from an open bioinformatics database and the tool to reveal molecular markers in plant proximal promoters [9].

Kim et al. (https://doi.org/10.3390/ijms252111784) used the Alphafold tool for structural prediction of laccase family proteins in the fungus *Auricularia auricula-judae* (white-rot fungus in wood) (Contribution 3). AlphaFold, a recently developed AI-based program, predicts protein structures with high accuracy from amino acid sequences [10]. The study by Kim et al. presents an application of AI tools [11] for a unique species important for biotechnology.

Riccardo Perriera and co-authors (https://doi.org/10.3390/ijms242015084) analyzed translational readthrough-inducing drugs (TRIDs) and their off-target effects on natural termination codons on TP53 and housekeeping gene expression (Contribution 4). This work highlights gene expression studies for fundamental biomedicine. Nonsense mutations cause several genetic diseases, such as cystic fibrosis, Duchenne muscular dystrophy, β-thalassemia, and Shwachman–Diamond syndrome. Nonsense mutations in gene coding regions introduce an in-frame premature termination codon in the mRNA transcript, resulting in the early termination of translation and the production of a truncated, nonfunctional protein. The absence of protein expression and the consequent loss of essential cellular functions are responsible for the severe phenotypes in the so-called genetic nonsense-related diseases (NRDs). This topic is under active development now. promising therapeutic approaches and emerging strategies for treating NRDs [12,13]. It considered both the use of small molecules to interfere with molecular mechanisms related to nonsense mutations, such as translational readthrough-inducing drugs or inhibitors of the nonsense-mediated decay pathway [14].

The following articles in this Special Issue discuss gene expression in model organisms (Contributions 5, 6, and 7). Zongchang Chen and co-authors (https://doi.org/10.3390/ijms25052506) studied the transcriptional control in bovine linked to the muscle formation and growth features of domestic animals (Contribution 5). This applied research for food meat production and quality revealed the role of non-SMC condensin I complex subunit G. Gene transcription in bovine models is being actively studied now [15,16].

The study by Qingpeng Shen and colleagues also focused on domestic animals’ growth (https://doi.org/10.3390/ijms242216205) (Contribution 6). The object of molecular studies was circular RNAs (CircRNAs) and their expression in multiple tissues of pigs. Thus, this study extends work by Chen and co-authors (https://doi.org/10.3390/ijms25052506) on domestic animals. Muscle development is a key factor influencing the growth performance of piglets. Recent gene expression studies to reveal microRNA in pigs continue this topic [17]. The topic of non-coding RNA and its annotation in model genomes based on integrated annotation and AI tools defines trends in gene expression studies [18]. An increasing number of non-coding RNAs (ncRNAs) are found to have roles in gene expression and cellular regulations [19].

Munkhzaya Byambaragchaa et al. (https://doi.org/10.3390/ijms25137282) produced single-chain recombinant Anguillid eel follicle-stimulating hormone analogs with high activity in *Cricetulus griseus* ovary DG44 cells (Contribution 7). The recent experimental work on equine follicle-stimulating hormone receptor presented effects of glycosylation sites on gene expression at the translation level complementing this research [20].

Igor V. Gorbenko and colleagues (https://doi.org/10.3390/ijms241311116) studied genome repeat structure in plants (Contribution 8). A novel DNA element called short interrupted repeat cassette (SIRC) was found throughout the *A. thaliana* nuclear genome. Contribution 8 continued previous studies on bioinformatics of plant stress response [21]. Plants have specific transcription control mechanisms for transposable elements, mobile DNA sequences that can amplify and change their chromosome position [22].

Thus, a variety of model species has to be studied by sequencing analysis methods exploring gene expression regulation topics, including RNA studies [23]. Machine learning models and artificial intelligence applications present new trends in gene expression studies [4,24,25]. Bioinformatics faces the challenge of integrating, aligning, modeling, and simulating data in a coherent fashion to gain deeper insights into complex biological systems, data retrieval [26], as it was discussed at the recent Integrative Bioinformatics conference [27,28]. The new insights on the problems associated with bioinformatics and discovering molecular mechanisms of gene expression in plant and animal models were presented in the Special Issues devoted to works on gene expression regulation starting from schools for young scientists [29], then after BGRS series conferences [1] in Frontiers in Genetics (https://www.frontiersin.org/research-topics/8383/bioinformatics-of-genome-regulation-and-systems-biology, accessed on 30 June 2025) and Frontiers in Plant Science (https://www.frontiersin.org/research-topics/54136/applications-of-artificial-intelligence-machine-learning-and-deep-learning-in-plant-breeding, accessed on 30 June 2025). Problems of plant bioinformatics will be discussed in the Special Issue titled “Plant Biology and Biotechnology: Focus on Genomics, Bioinformatics and AI” (https://www.mdpi.com/journal/ijms/special_issues/1D646O0T7V, accessed on 30 June 2025). Problems of gene expression from a biophysical point of view will be presented during the open call of the Biophysical Reviews journal (“Modern Biophysical Methods: Insights from the Russian Autumn School on Biophysics in Kazan 2024”) (https://link.springer.com/collections/ifbbihjicj, accessed on 30 June 2025).

This topical Special Issue “Bioinformatics of Gene Regulations and Structure–2025” (https://www.mdpi.com/journal/ijms/special_issues/3I56FG1O3A, accessed on 30 June 2025), continues the series of collective works on gene expression. We hope that readers find these materials to be interesting and stimulating, and we will continue to collect papers on bioinformatics of gene expression for ongoing topical issues.
